# Clinton Medical Society

**Published:** 1866-08

**Authors:** C. Goodbrake, B. K. Shurtleff

**Affiliations:** Pres’t.; Sec’y.


### CLINTON MEDICAL SOCIETY.

At a meeting of the Clinton Institute of Medicine, held on the 19th day of June, 1866, the following preamble and resolutions were, on motion of Dr. John A. Edmiston, unanimously adopted:—

*Whereas*, We, the physicians of Clinton, Ill., having been, from time to time, strongly solicited by the agent of J. R. Nichols & Co., manufacturing chemists, of Boston, Mass., to prescribe and recommend their chemicals, tinctures, and mixtures; and,

*Whereas*, We have seen advertisements in the public newspapers, wherein Nichols & Co.'s preparations are recommended and puffed to the public the same as other quack nostrums; therefore, be it

*Resolved*, That the course pursued by James R. Nichols & Co., for the purpose of introducing their preparations, as shown by their advertisement in the *Chicago Daily Republican* of June 15th, 1866, recommending as a popular medicine for general use, their preparation of the syrup of sarsaparilla with iodide of lime, is not only detrimental to the interest of the public, as well as the profession, but a violation of their assurances, that they would resort to no unfair means.

While they confined themselves to the legitimate object of furnishing the profession their preparations in a pure and reliable form, we believed it a worthy object, and gave them a hearty support; but, by this endeavor to vend one of their preparations on the same plan as the many patent humbugs of the day, they lose our confidence, and, if persisted in, we will consider it good and sufficient cause for their rejection from our practice, altogether.

*Resolved*, That the Secretary be instructed to forward a copy of these resolutions, signed by the members of this Institute, to James R. Nichols & Co., and, also, a copy to each of the Chicago medical journals.

C. GOODBRAKE, M.D., *Pres't.*

B. K. SHURTLEFF, M.D., *Sec'y.*

